# Life expectancy and human capital: New empirical evidence

**DOI:** 10.1002/hec.4626

**Published:** 2022-10-31

**Authors:** Trung V. Vu

**Affiliations:** ^1^ School of Business and Economics Loughborough University Loughborough UK

**Keywords:** cross‐sectional dependence, human capital, life expectancy, misspecification, parameter heterogeneity

## Abstract

This paper re‐examines a well‐established hypothesis postulating that life expectancy augments incentives for human capital accumulation, leading to global income differences. A major distinguishing feature of the current study is to estimate heterogeneous panel data models under a common factor framework, which explicitly accounts for parameter heterogeneity, unobserved common factors (UCFs), and variables' non‐stationarity. In sharp contrast to most previous studies, I find that the impact of health improvements on human capital accumulation turns out to be imprecisely estimated at conventionally accepted levels of statistical significance. I demonstrate that conventional estimates of the educational returns to rising longevity are derived from estimating misspecified models at least partially due to parameter heterogeneity and the presence of UCFs.

## 1 INTRODUCTION

The elusive quest for the causes of comparative prosperity across the world remains a persistent feature of ongoing debate in mainstream economics. A major contribution to this line of inquiry postulates that health capital is a key determinant of global income differences. This viewpoint, in particular, is motivated by an early and influential paper by Preston (1975) that documents a positive association between human longevity and economic prosperity in a cross‐sectional framework. Subsequent attempts at empirical estimation lend strong credence to the growth‐enhancing effect of population health (e.g., Aghion et al., 2011; Bloom et al., 1998; Bloom et al., 2004; Bloom & Williamson, 1998; Cervellati & Sunde, 2011; Knowles & Owen, 1995, 1997; Lorentzen et al., 2008). Divergent from this consensus, Acemoglu and Johnson (2007) find that exogenous variation in life expectancy, driven by the international epidemiological transition after the Second World War, exerts a statistically insignificant or even negative effect on economic growth across countries.

Existing explanations of the positive effect of life expectancy on economic performance primarily rest upon the conventional wisdom that health improvements strengthen human capital investments. The underlying idea is that increased longevity augments motivations to accumulate knowledge by increasing the horizon over which investments in education are discounted (Becker, 1994; Ben‐Porath, 1967; Heckman, 1976).^1^ The theoretical justification for this proposition is further developed by Hazan (2009) and Hansen and Lønstrup (2012). On the empirical side, the existing literature is far from conclusive when it comes to establishing the hypothesis that rising longevity helps enhance the incentive to acquire better human capital skills. Several studies, for instance, document a positive association between longevity, early childhood health, and years of schooling (Boucekkine et al., 2002; Currie et al., 2010). Furthermore, Zhang and Zhang (2005) show that rising longevity significantly increases secondary school enrollment. By contrast, Acemoglu and Johnson (2007) find that life expectancy has a weak or statistically insignificant influence on years of schooling. Hazan (2012) indicates that life expectancy at birth positively affects schooling, but life expectancy at age 5 is uncorrelated with variations in schooling. Hansen (2013b) reveals that changes in life expectancy as a result of the epidemiological transition in the late 1940s are associated with significant improvements in human capital across the globe. More recently, Hoque et al. (2019) employ survey data from 147 countries and reveal that increased life expectancy has a positive and statistically significant influence on lifetime completed years of schooling.^2^

In addition, several studies provide suggestive evidence of heterogeneity in the impact of health improvements on economic growth and human capital across countries. For example, Cervellati and Sunde (2011) demonstrate that the contribution of life expectancy to shaping cross‐country differences in income per person is non‐linear depending on the onset of the demographic transition. More specifically, life expectancy positively affects growth among post‐transitional countries, but rising longevity is detrimental to economic performance before the demographic transition (Cervellati & Sunde, 2011). In a similar vein, Cervellati and Sunde (2015) find evidence of heterogeneity in the impact of life expectancy on education at different stages of demographic development. Other scholars postulate that the relationship between life expectancy and education hinges upon individuals' preferences, labor market characteristics, and the supply of labor (Sánchez‐Romero et al., 2016; Strulik & Werner, 2016).

A conventional approach to examining the relationship between life expectancy and human capital across countries is to estimate cross‐sectional, and/or long difference and decade(s)‐averaged panel data models.^3^ It is noteworthy that the estimators widely adopted in existing studies implicitly carry a relatively restrictive assumption of homogeneity in the effect of life expectancy on human capital. In other words, these studies rely on empirical models that consider the health‐education nexus as *common* across the globe. As mentioned previously, the existing literature is suggestive of heterogeneity in the impact of human longevity on education across countries at different stages of the demographic transition and economic development (Cervellati & Sunde, 2011, 2015; Sánchez‐Romero et al., 2016; Strulik & Werner, 2016). Nevertheless, previous studies have predominantly provided empirical estimates of *the cross‐country average effect* of health on human capital accumulation. It is important to note that the extent to which human capital accumulation responds to health improvements presumably differs substantially across countries due to considerable heterogeneity in institutions, cultures, technologies, and socio‐economic development. From an econometric perspective, distinguishing between a common parameter based on pooled models ($\hat{\beta }$) and a heterogeneous parameter derived from heterogeneous parameter models is central to securing reliable inference on the health‐education relationship (see Pesaran & Smith, 1995). From the perspective of policymakers, effective policy interventions in individual countries critically require a comprehensive understanding of heterogeneity in the influence of rising longevity on human capital across the world. However, previous studies merely suggest that life expectancy on average leads to better human capital but are not useful for designing relevant country‐specific policies. Consistent with the above discussion, the arguments of Cervellati and Sunde (2011, 2015), Sánchez‐Romero et al. (2016), and Strulik and Werner (2016) motivate the exploration of heterogeneity in the impact of life expectancy on human capital in a cross‐country framework.^4^

Identification of the effect of life expectancy on education also requires attention to unobserved common factors (UCFs) that simultaneously affect national health status and human capital accumulation. This concern at least partially stems from the interdependence between world economies. For example, countries could suffer from global common shocks or are subject to the cross‐border diffusion of health technologies and knowledge, thereby affecting a country's health and educational outcomes. However, it is very difficult to identify, measure, and include numerous UCFs in standard regression models. This is because including these common factors in econometric models requires identifying numerous confounding factors, and their unobserved nature makes it inconvenient to measure and identify properly. In addition, incorporating a variety of control variables in the regression may impose substantial constraints on the feasible degrees of freedom. Another concern is that the unknown relationship between UCFs and human capital could result in potential functional form misspecification if they were to be included in the regression. Thus, UCFs generally enter econometric models in the form of disturbance terms in previous studies. Furthermore, earlier studies, to my knowledge, largely ignore the potential non‐stationarity of life expectancy at birth and human capital when investigating the educational returns to health improvements.

Motivated by the above concerns, I re‐estimate the effect of life expectancy on human capital accumulation across countries considering the importance of parameter heterogeneity, variables' non‐stationarity, and UCFs. To this end, I construct a balanced panel dataset covering 79 countries over the period from 1964 to 2015. A key distinguishing feature of this study stems from empirical attempts at addressing the above econometric issues. To preview the main findings, I find that life expectancy has a sizable and significant positive effect on human capital accumulation when using conventional panel estimators that do not account for UCFs, variables' non‐stationarity, and parameter heterogeneity. This is in line with most previous studies reviewed earlier. However, the impact of life expectancy on human capital turns out to be imprecisely estimated at conventionally accepted levels of statistical significance once I explicitly account for parameter heterogeneity and UCFs. Conducting several residual‐based diagnostic tests, the preferred estimates provide suggestive evidence that life expectancy, on average, has no statistically significant impact on human capital accumulation. On this basis, I argue that the findings established in the existing literature are derived from estimating misspecified models partly due to the presence of UCFs and parameter heterogeneity.

The empirical approach of this study builds upon an emerging body of research revisiting some well‐established hypotheses by applying the common factor framework of Pesaran (2006). For example, Eberhardt et al. (2013) re‐estimate the Griliches‐type production function that evaluates private returns to R&D expenditure, and find that spending on R&D has no statistically significant influence on outputs when controlling for spillover effects across countries. In an attempt to re‐consider the contribution to output of infrastructure capital, Calderón et al. (2015) suggest that estimators ignoring UCFs and parameter heterogeneity considerably inflate the estimated coefficients. Baltagi and Moscone (2010) indicate that the income elasticity of health care spending is much smaller after ruling out the potential bias of UCFs. In addition, Dissanayake (2016) demonstrates that UCFs are of importance when estimating the transmission elasticity of international food price shocks to domestic markets. In line with these studies, this paper contributes to the existing literature by employing the common factor approach to re‐estimate the effect of longevity on human capital accumulation.

The rest of the paper is organized as follows. Section 2 discusses data and empirical methods. Sections 3 and 4 contain the main results and robustness analyses, respectively. Section 5 presents some concluding remarks.

## 2 EMPIRICAL STRATEGY

### 2.1 Variables and data

To revisit the relationship between life expectancy and human capital accumulation, I construct a balanced panel dataset covering 79 countries between 1964 and 2015. The main variable of interest is life expectancy at birth, taken from the World Bank's World Development Indicators (http://wdi.worldbank.org/). The dependent variable is the human capital index obtained from the Penn World Table 9.1 (Feenstra et al., 2015). This indicator measures the quality of human capital based on a “Mincerian” combination of years of schooling and returns to education (Feenstra et al., 2015).^5^

### 2.2 Model specification

To compare and contrast my findings with conventional estimates in earlier studies, I adopt several panel estimators with different assumptions about data properties, parameter heterogeneity, and the error term. The preferred model is chosen based on some residual‐based diagnostic tests that help identify model misspecification, following Eberhardt et al. (2013) and Eberhardt and Teal (2020). The baseline model specification can be expressed as follows:

(1) $${\ln\left(\text{HC}\right)}_{it}=\beta_{i}\ln{\left(\text{LE}\right)}_{it}+\mu_{i}+\tau_{i}f_{t}+\varepsilon_{it}$$

where ln represents the logarithmic transformation of variables. HC and LE are the human capital index and life expectancy at birth, respectively. i and t denote country i and year t, respectively (N=79). I use annual data from 1964 to 2015 (T=52). Unobserved country‐specific factors are captured by μ. $f_{t}$ stands for a set of UCFs with country‐specific factor loadings $\tau_{i}$. β reflects the estimated effect of life expectancy on human capital accumulation. The subscript i on β allows it to differ across cross‐sectional units (parameter heterogeneity).

### 2.3 Estimation methods

Conventional panel estimation methods include pooled OLS (POLS) and fixed‐effects (FE) estimators, which have been widely used to explore the health‐education nexus. These estimation techniques require that variables follow stationary processes, the violation of which may yield a spurious relationship among regressors. Although FE models allow the intercept estimates to vary across countries, the estimated slope coefficients, by construction, are identical across cross‐sectional units in the sample (Wooldridge, 2010). Furthermore, the presence of UCFs invalidates the OLS estimates because the error term is correlated across cross‐sectional units. Therefore, these conventional estimators may yield biased and inconsistent estimates if the aforementioned restrictive assumptions are not satisfied (Chudik et al., 2011; Pesaran & Smith, 1995).

This paper first examines data properties carefully by applying several panel unit‐root tests. For ease of comparison, I implement three non‐stationarity tests with different assumptions about cross‐sectional dependence (CSD). The main conclusion, however, is based on the “second‐generation” panel unit‐root test (CIPS) of Pesaran (2007) because it is robust to CSD. As discussed previously, the unit‐root tests can also be used to check for the stationarity of residuals, which helps detect model misspecification (Eberhardt et al., 2013; Eberhardt & Teal, 2020). Next, I perform a test of parameter homogeneity following Pesaran and Yamagata (2008). In the case of slope heterogeneity, the mean group estimator developed by Pesaran and Smith (1995) is used to obtain consistent estimates. The basic idea of this approach is to estimate a single linear regression for each country. By doing so, the estimated effect of rising longevity on human capital is allowed to differ across cross‐sectional units. Next, the estimated average effects are calculated as averages across countries.

A major concern when exploring the relationship between life expectancy and human capital stems from potential endogeneity bias. This could arise because UCFs may be correlated with LE and HC. Furthermore, reverse causality, if it exists, may also invalidate inference on the education‐health nexus. As such, conventional panel estimators may yield a spurious relationship between longevity and human capital accumulation (Phillips & Sul, 2003; Sarafidis & Robertson, 2009). A common identification strategy is to exploit exogenous variation in life expectancy that helps explain human capital accumulation (Acemoglu & Johnson, 2007; Hansen, 2013b). Another approach relies on using internal instruments, which are lags of endogenous regressors (Arellano & Bond, 1991; Blundell & Bond, 1998). These estimation methods, however, can be biased and inconsistent if any of the assumptions of slope homogeneity, stationarity, and cross‐sectional independence are violated. Furthermore, the identification approach of Acemoglu and Johnson (2007) is not applicable in this context because I exploit within‐country variations in the data between 1964 and 2015.

To mitigate endogeneity concerns, I first conduct the test of CSD developed by Pesaran (2015). The aim is to check for the correlation of the unobserved error term across countries. Indeed, macroeconomic variables are generally interrelated across countries (Chudik et al., 2011; Pesaran, 2015). I interpret the rejection of cross‐sectional independence as an indication of UCFs, consistent with Calderón et al. (2015), Dissanayake (2016), Eberhardt et al. (2013), and Eberhardt and Teal (2020). To account for UCFs, I apply the common factor framework of Pesaran (2006). In this regard, endogeneity concerns are partly addressed by controlling for the correlation between UCFs ($f_{t}$) and LE. This method is implemented by incorporating cross‐sectional averages for both dependent and independent variables in the regression (Pesaran, 2006). An advantage of applying the common factor framework is that it does not require a priori knowledge about the underlying relationship between UCFs and the main variable of interest. By using the mean group estimator under the common factor framework, I allow the estimated coefficients to differ across countries (parameter heterogeneity). In particular, the averaged coefficient will be computed as follows: $\hat{\beta }=N^{-1}\sum_{i=1}^{N}{\hat{\beta }}_{i}$.

Table 1 contains a summary of different panel data estimators adopted in this paper. Estimation methods in the left column assume homogeneous parameters ($\beta_{i}=\beta$ for all i=1,…,N) and stationary variables. They also require cross‐sectional independence (except for the common correlated effects pooled estimator—CCEP). These estimates are reported for the purpose of comparison. The mean group type estimators, listed in the right column, relax the restrictive assumption of parameter homogeneity. Additionally, the common factor framework (CCE) of Pesaran (2006) is adopted to address UCFs. The Online Appendix provides more detailed discussions about the above estimators and diagnostic tests.

**TABLE 1:** Assumptions of different panel estimators

		Parameter homogeneity	
		Yes	No
Cross‐sectional independence	Yes	POLS, 2FE, FD	MG
	No	CCEP	CMG, AMG

*Note*: POLS is the pooled ordinary least squares estimator. FD is the first‐difference estimator. 2FE stands for the two‐way fixed effects estimator, which accounts for unobserved country‐ and year‐specific factors. The POLS, 2FE, and FD estimators assume slope homogeneity, and do not account for UCFs. The assumption of parameter homogeneity can be relaxed by using the mean group estimator (MG) developed by Pesaran and Smith (1995). Pesaran (2006) and Bond and Eberhardt (2009) propose the use of common correlated effect mean group (CMG) and augmented mean group (AMG) to account for slope heterogeneity and cross‐sectional dependence. CCEP is the POLS estimator under the common factor framework that considers cross‐sectional correlation but assumes parameter homogeneity. The Online Appendix contains a more detailed description of these estimators.

## 3 RESULTS

### 3.1 A preliminary analysis

I begin the analysis by checking for the stationarity of variables (Table 2). The results of “first‐generation” panel unit‐root tests are presented in Panels A and B of Table 2. I also use the “second‐generation” panel unit‐root test of Pesaran (2007) that explicitly accounts for cross‐sectional correlation (Panel C, Table 2). Accordingly, the null hypothesis of non‐stationarity is rejected in most cases when I perform the different tests for variables in (log) levels. This suggests that LE and HC follow non‐stationary processes. By contrast, the variables broadly become stationary when I transform them into first differences. This provides evidence that they may be integrated of order one. Furthermore, I conduct the CSD test of Pesaran (2015) to check for the presence of UCFs. Accordingly, I find strong evidence of cross‐sectional correlation (Table 3). The results suggest that conventional estimators that fail to account for CSD may suffer from endogeneity bias induced by the correlation between UCFs and LE. To check for the possibility that the educational returns to health improvements are heterogeneous across countries, I implement the slope homogeneity test developed by Pesaran and Yamagata (2008). The results in Table 4 lend credence to the violation of the parameter homogeneity assumption. This necessitates using the mean group type estimators to obtain reliable estimates of average effects across countries.

**TABLE 2:** First‐ and second‐generation panel unit‐root tests

	Variables in levels		Variables in first differences		
Lags	ln (LE)	ln (HC)	Lags	ln (LE)	ln (HC)
Panel A. Im et al.’s (2003) (IPS) panel unit‐root test					
0	10.58 [1.00]	9.66 [1.00]	0	−9.99 [0.00]	−0.46 [0.32]
1	−18.16 [0.00]	−2.16 [0.02]	1	−3.02 [0.00]	−0.22 [0.41]
2	−10.15 [0.00]	−0.95 [0.17]	2	−4.82 [0.00]	−1.65 [0.05]
3	−4.07 [0.00]	0.42 [0.66]	3	−8.98 [0.00]	−2.90 [0.00]
4	3.85 [0.99]	1.18 [0.88]	4	−4.57 [0.00]	−6.80 [0.00]
Panel B. Maddala and Wu's (1999) Fisher panel unit‐root test					
0	507.27 [0.00]	277.89 [0.00]	0	1333.13 [0.00]	149.63 [0.67]
1	1750.89 [0.00]	222.03 [0.00]	1	2601.11 [0.00]	149.50 [0.67]
2	343.44 [0.00]	155.61 [0.54]	2	304.21 [0.00]	163.82 [0.36]
3	396.17 [0.00]	142.93 [0.80]	3	299.54 [0.00]	198.26 [0.02]
4	214.91 [0.00]	137.79 [0.88]	4	274.49 [0.00]	320.72 [0.00]
Panel C. Pesaran's (2007) (CIPS) panel unit‐root test					
0	1.47 [0.93]	19.22 [1.00]	0	−5.17 [0.00]	−0.61 [0.27]
1	−16.36 [0.00]	−0.17 [0.43]	1	−30.47 [0.00]	−0.68 [0.25]
2	7.59 [1.00]	2.13 [0.98]	2	−2.05 [0.02]	−1.79 [0.04]
3	−1.31 [0.10]	3.18 [1.00]	3	−1.74 [0.04]	−3.11 [0.00]
4	1.98 [0.98]	4.31 [1.00]	4	−2.24 [0.01]	−8.12 [0.00]

*Note*: This table reports the results of panel unit‐root tests for variables in levels and first differences. CIPS is the second‐generation panel unit‐root test that accounts for CSD (Pesaran, 2007). The null hypothesis of these tests is that variables are non‐stationary.

**TABLE 3:** Cross‐sectional dependence (CSD) test

	ln (LE)	ln (HC)		ln (LE)	ln (HC)
Panel A. Variables in levels			Panel B. Variables in first differences		
Average ρ	0.865	0.960	Average ρ	0.137	0.099
Average |ρ|	0.889	0.960	Average |ρ|	0.296	0.372
CD‐test	342.78	380.70	CD‐test	54.14	39.10
*p*‐value	0.000	0.000	*p*‐value	0.000	0.000
Panel C. Pooled AR (2) regressions			Panel D. Country AR (2) regressions		
Average ρ	0.116	0.017	Average ρ	0.050	0.058
Average |ρ|	0.333	0.272	Average |ρ|	0.206	0.270
CD‐test	45.66	6.67	CD‐test	19.45	22.91
*p*‐value	0.000	0.000	*p*‐value	0.000	0.000

*Note*: This table reports the results of Pesaran's (2015) test of weak cross‐sectional dependence. I report the average and absolute value of correlation coefficients between N(N−1) pairs of country correlation. CD stands for the test statistic values under the null hypothesis of no cross‐sectional correlation. Thus, the rejection of the test indicates cross‐sectional dependence. I apply the test for variables in levels and first differences in Panels A and B, respectively. I further check for CSD of the error term of the autoregressive regressions in Panel C and Panel D. A pooled OLS estimator is used in Panel C with year dummies included as controls. I use the mean group estimator in Panel D with time trend included. See also the Online Appendix for a more detailed description.

**TABLE 4:** Parameter homogeneity test

Unobserved common factors	(1)	(2)	(3)
	Ignoring	Demeaning	CCE
Delta statistic [*p*‐value]	169.368 [0.000]	151.768 [0.000]	−232.112 [0.000]
Delta adjusted statistic [*p*‐value]	174.476 [0.000]	156.344 [0.000]	−228.896 [0.000]

*Note*: This table reports the results of the slope homogeneity test developed by Pesaran and Yamagata (2008) under the null hypothesis of homogeneous parameters. I report the standardized delta test statistic and the biased adjusted test statistic (Pesaran & Yamagata, 2008). The test is performed by estimating and comparing two models. The first model is estimated using the FE estimator that assumes slope homogeneity. The second model is estimated for each single country, assuming slope heterogeneity. In columns (2) and (3), the issue of cross‐sectional dependence is addressed by subtracting cross‐sectional mean values (demeaning) or applying the common factor framework (CCE). See also the Online Appendix for a more detailed description.

### 3.2 Main findings

This section presents empirical estimates of the effect of life expectancy on human capital using the panel estimators listed in Table 1. I first estimate Equation (1) assuming homogeneous parameters across countries. Next, I proceed to the results of heterogeneous parameter estimates. Following Eberhardt et al. (2013) and Eberhardt and Teal (2020), I report the results of several residual‐based diagnostic tests to detect model misspecification. This helps in choosing the preferred estimates and allows for the comparison of results derived from models that do and do not account for UCFs.

#### 3.2.1 Homogeneous parameter estimates

Table 5 reports homogenous parameter estimates of the effect of life expectancy on human capital accumulation across countries. As shown in columns (1) to (3), the estimated coefficient on log of life expectancy is positive and statistically significant at the 1% level. I pool data across countries and control for year‐specific factors; the POLS estimates are suggestive a sizable influence on education in response to rising longevity. However, the estimated coefficient becomes much smaller when I control for unobserved country‐specific factors using the 2FE estimator. Accordingly, a one‐percent increase in life expectancy at birth is associated with an approximately 0.3‐percent increase in the human capital index. Turning to column (3) of Table 5, a significant positive effect is observed using the first‐difference (FD) estimator. It is noteworthy that taking first differences removes much cross‐sectional and time‐series variation in the data, making it hard to draw inference on the long‐run effect of health improvements on human capital.^6^

**TABLE 5:** Homogeneous parameter estimates of the effect of life expectancy on human capital

Estimator	(1)	(2)	(3)	(4)
	POLS	2FE	FD	CCEP
Dependent variable is log of human capital index				
Log of life expectancy	1.596***	0.307***	0.084***	−0.007
	(0.022)	(0.026)	(0.018)	(0.026)
Year FE	Yes	Yes	Yes	Yes
Country FE	No	Yes	No	Yes
Observations	4108	4108	4029	4108
Number of countries	79	79	79	79
Diagnostics				
RMSE	0.17	0.06	0.01	0.02
CSD	0.00	0.12	0.17	0.00
CIPS	0.79	1.00	0.33	1.00
AR (1)	0.00	0.00	0.00	0.00
AR (2)	0.00	0.00	0.00	0.00

*Note*: This table reports empirical estimates of the effect of life expectancy on human capital accumulation across countries. In columns (1) to (3), I use conventional panel data estimators assuming parameter homogeneity and cross‐sectional independence to estimate the benchmark model. In column (4), I account for cross‐sectional dependence by performing pooled OLS regression under a common factor framework. An intercept is included in all the regressions but is omitted for brevity. CSD denotes *p*‐value of the test for weak cross‐sectional dependence (Pesaran, 2015). CIPS is *p*‐value of the second‐generation panel unit‐root test of Pesaran (2007) using three lags, a constant and trend. AR stands for *p*‐value of the test of no residual serial correlation (Arellano & Bond, 1991). RMSE is the root mean squared error—a measure of the goodness of fit. Robust standard errors in parentheses.

Abbreviations: CCEP, common correlated effects pooled; FD, first‐difference.

*** denotes statistical significance at the 1% level.

I test for the stationarity of the residuals of models based on the panel unit‐root test of Pesaran (2007). Furthermore, UCFs can also be detected by conducting the CSD test of Pesaran (2015). The evidence of serial correlation in the error terms can be tested, using the autocorrelation test of Arellano and Bond (1991). The results indicate that the homogeneous parameter models are seriously misspecified given the evidence of non‐stationary and serially correlated residuals (Table 5). The CSD test suggests that the POLS regression may suffer from bias and inconsistency induced by the presence of UCFs. The null of cross‐sectional independence is rejected at conventionally accepted levels of statistical significance. Overall, the POLS, 2FE, and FD estimates are consistent with previous studies establishing a positive and significant effect of life expectancy on human capital accumulation (Hansen, 2013b; Zhang & Zhang, 2005). However, these estimates do not necessarily provide a valid basis for statistical inference due to parameter heterogeneity and UCFs. Turning to column (4) of Table 5, the effect of UCFs is controlled for using the common factor framework of Pesaran (2006). Interestingly, the effect of life expectancy on human capital is imprecisely estimated at conventional levels of statistical significance when I account for UCFs. The CSD test, however, suggests that the CCEP estimator is not effective at removing UCFs because the null of cross‐sectional independence of the residuals is rejected. The model is also seriously misspecified with non‐stationary, serially, and cross‐sectionally correlated residuals.

#### 3.2.2 Heterogeneous parameter estimates

I present heterogeneous parameter estimates in Table 6. In column (1), I estimate the benchmark model using the mean group estimator that allows the estimated coefficient to vary across cross‐sectional units. The results suggest that life expectancy, on average, has no statistically significant effect on human capital. However, the MG estimator does not explicitly control for UCFs, evidenced by the result of the CSD test. A common approach in the panel time‐series literature is to demean the data to reduce CSD. The estimated coefficient of life expectancy is statistically significant and positive when I apply the cross‐sectional demeaned mean group (CDMG) estimator (column 2). Nevertheless, the CDMG model is misspecified with a non‐stationary residual and failure to control for CSD. Next, I employ the CMG estimator of Pesaran (2006) to estimate Equation (1). Accordingly, I find no evidence of misspecification of the CMG model (column 3). In particular, the error term is stationary and not serially correlated while I fail to reject the null of cross‐sectional independence. Hence, the CMG estimates are preferred. They reveal that health improvements on average have no statistically significant impact on human capital accumulation, which is in contrast to most previous studies. The lack of statistical significance remains largely unchanged when I apply the augmented mean group estimator (AMG) of Bond and Eberhardt (2009). This estimation method is developed based on the CMG estimator in which the common factor is treated as a “common dynamic process”. To summarize, I find that the impact of life expectancy on human capital accumulation is imprecisely estimated and statistically insignificant at conventionally accepted levels when the econometric model is appropriately specified.

**TABLE 6:** Heterogeneous parameter estimates of the effect of life expectancy on human capital

Estimator	(1)	(2)	(3)	(4)
	MG	CDMG	CMG	AMG
Dependent variable is log of human capital index				
Log of life expectancy	0.166	0.635***	−0.012	0.154
	(0.133)	(0.154)	(0.095)	(0.144)
Trend included	Yes	No	Yes	Yes
Observations	4108	4108	4108	4108
Number of countries	79	79	79	79
Diagnostics				
RMSE	0.02	0.04	0.01	0.02
CSD	0.00	0.00	0.34	0.00
CIPS	0.00	0.17	0.00	0.00

*Note*: This table reports empirical estimates of the effect of life expectancy on human capital accumulation. More specifically, I estimate the benchmark model by using several panel data estimators that account for cross‐sectional dependence and parameter heterogeneity. CDMG stands for the cross‐sectional demeaned mean group estimator. Robust standard errors in parentheses.

Abbreviation: RMSE, root mean squared error.

*** denotes statistical significance at the 1% level.

#### 3.2.3 Reconciling the results

This paper revisits the relationship between life expectancy and human capital by using different panel estimation methods. I find a significant positive and sizable effect of longevity on education when using conventional panel estimators assuming slope homogeneity and ignoring UCFs. Performing several residual‐based diagnostic tests, I demonstrate that the significant positive effect of life expectancy on human capital is derived from estimating misspecified models, evidenced by the non‐stationary, serially correlated, and cross‐sectionally dependent residuals. When I explicitly account for UCFs and parameter heterogeneity, the educational returns to rising longevity appear at best modest. This is in line with the study by Acemoglu and Johnson (2007) positing that rising longevity leads to population growth, and hence retards resources for investments in obtaining knowledge and education. For this reason, life expectancy has no statistically significant impact on human capital accumulation (Acemoglu & Johnson, 2007). My findings complement previous studies in demonstrating that UCFs and parameter heterogeneity cannot be ignored when investigating the impact of life expectancy on human capital accumulation.

#### 3.2.4 The role of demographic and economic development

As discussed earlier, several scholars document evidence of heterogeneity in the impact of life expectancy on human capital accumulation at different stages of demographic and economic development. Cervellati and Sunde (2015) posit that rising longevity leads to a significant increase in schooling only after the onset of the demographic transition when countries typically experience a large decrease in population growth and birth rates. It is argued that the main results can hide substantial differences in the educational returns to health improvements across countries at different stages of the demographic transition. To address this concern, I replicate the main analysis using two different samples of pre‐ and post‐transitional countries. Following Cervellati and Sunde (2011), post‐transitional countries include ones with life expectancy greater than the conventional threshold of 50 years in 1963. Table 7 reports the 2FE and CMG estimates of the impact of LE on HC for pre‐ and post‐transitional countries. The 2FE results reveal that rising longevity leads to significantly higher levels of educational attainment for post‐transitional economies, but the economic and statistical significance of the positive impact of life expectancy on education is much smaller for countries during early stages of demographic development (Columns 1 and 2). This provides support for the findings of Cervellati and Sunde (2015). However, the estimated coefficient on log of life expectancy turns out to be imprecisely estimated at conventionally accepted levels of statistical significance when using the mean‐group type estimator under a common factor framework (Columns 3 and 4). In Table 8, I also present 2FE and CMG estimates of the effect of life expectancy on human capital for countries at different levels of economic development, following the World Bank's classification. The preferred CMG estimates are statistically insignificant at conventional levels, although the 2FE results are suggestive of the heterogeneous impact of longevity on education. Consistent with the main findings, the results in Tables 7 and 8 suggest that securing reliable inference on the health‐education nexus critically requires attention to parameter heterogeneity and the presence of UCFs.

**TABLE 7:** The effect of life expectancy on human capital for countries at different stages of the demographic transition

	2FE		CMG	
	(1)	(2)	(3)	(4)
Estimator	Pre‐transitional countries	Post‐transitional countries	Pre‐transitional countries	Post‐transitional countries
Dependent variable is log of human capital index				
Log of life expectancy	0.068**	0.558***	−0.092	0.019
	(0.031)	(0.066)	(0.146)	(0.093)
Year FE	Yes	Yes	No	No
Country FE	Yes	Yes	No	No
Trend included	No	No	Yes	Yes
Observations	1560	2548	1560	2548
Number of countries	30	49	30	49
Diagnostics				
RMSE	0.06	0.06	0.01	0.01
CSD	0.00	0.00	0.00	0.00
CIPS	1.00	0.98	0.04	0.00
AR (1)	0.00	0.00		
AR (2)	0.00	0.00		

*Note*: This table replicates the main analysis using two samples of pre‐ and post‐transitional countries, characterized by different levels of demographic development. Robust standard errors in parentheses.

Abbreviation: RMSE, root mean squared error.

*** and ** denote statistical significance at the 1% and 5% levels, respectively.

**TABLE 8:** The effect of life expectancy on human capital for countries at different levels of economic development

Estimator	2FE				CMG				
	(1)	(2)	(3)	(4)	(5)	(6)	(7)	(8)
	Low‐income countries	Lower‐middle‐income countries	Upper‐middle‐income countries	High‐income countries	Low‐income countries	Lower‐middle‐income countries	Upper‐middle‐income countries	High‐income countries
Log of life expectancy	−0.048	−0.092***	0.459***	1.655***	−0.006	−0.380	0.248	−0.349
	(0.106)	(0.020)	(0.041)	(0.096)	(0.121)	(0.183)	(0.341)	(0.186)
Year FE	Yes	Yes	Yes	Yes	No	No	No	No
Country FE	Yes	Yes	Yes	Yes	No	No	No	No
Trend included	No	No	No	No	Yes	Yes	Yes	Yes
Observations	312	1196	1196	1404	312	1196	1196	1404
Number of countries	6	23	23	27	6	23	23	27
Diagnostics								
RMSE	0.08	0.05	0.06	0.04	0.01	0.01	0.01	0.01
CSD	0.00	0.00	0.02	0.02	0.00	0.02	0.00	0.82
CIPS	1.00	1.00	0.96	0.72	0.03	0.04	0.00	0.00
AR(1)	0.00	0.00	0.00	0.00				
AR(2)	0.00	0.00	0.00	0.00				

*Note*: This table replicates the main analysis using different samples of countries at various stages of economic development as classified by the World Bank. Robust standard errors in parentheses.

Abbreviation: RMSE, root mean squared error.

*** denotes statistical significance at the 1% level.

## 4 ROBUSTNESS

### 4.1 Estimating a dynamic model

One may argue that drawing inference on the long‐run relationship between life expectancy and human capital based on a static model may misinterpret the short‐run fluctuations as the long‐run impacts. To address this concern, I specify the following simple error correction model:

(2) $$\Delta {\ln\left(\text{HC}\right)}_{it}=\rho_{i}{\ln\left(\text{HC}\right)}_{i,t-1}+\beta_{i}\Delta {\ln\left(\text{LE}\right)}_{it}+\gamma_{i}{\ln\left(\text{LE}\right)}_{i,t-1}+\mu_{i}+\tau_{i}\Delta f_{t}+\varepsilon_{it}$$

where β captures the short‐run effect of life expectancy on human capital. ρ reflects the speed of convergence to the long‐run equilibrium. The long‐run impact of longevity on human capital is calculated as follows: $\theta_{i}=-\gamma_{i}/\rho_{i}$. Consistent with the main analysis, I also estimate Equation (2) using the panel estimators listed in Table 1.

I report the estimation results for the dynamic model specification in Table 9. The homogenous parameter estimates are presented in columns (1) and (2). The remaining columns report the estimates that explicitly control for UCFs and relax the assumption of parameter homogeneity. I report several residual‐based diagnostic tests to detect model misspecification and select the preferred estimates. Accordingly, the long‐run estimated coefficient of life expectancy has a positive sign and is statistically significant at the 1% level when using the POLS estimator. In contrast, the short‐run estimate is statistically insignificant at conventionally accepted levels. However, the results of diagnostic tests suggest that the POLS model is misspecified because of the non‐stationary and cross‐sectionally correlated residuals. Turning to column (2) of Table 9, the short‐ and long‐run coefficients on log of life expectancy are very imprecisely estimated. The panel unit‐root test applied to the residuals suggests that the 2FE model is misspecified with non‐stationary unobserved components. Furthermore, I employ the MG and CMG estimators to estimate Equation (2) (Columns 3 and 4). The estimation results indicate that life expectancy has no statistically significant influence on human capital when I account for UCFs and slope heterogeneity. Diagnostic tests suggest that the preferred estimates are those in column (4). In particular, the CMG model has the smallest value of root mean squared error while there is no evidence of CSD and non‐stationarity in the residual. By estimating a simple error correction model, I consistently find that ignoring UCFs and slope heterogeneity produces a sizable and statistically significant impact of life expectancy on human capital. These results are in line with the core findings.^7^

**TABLE 9:** The effect of life expectancy on human capital, dynamic model estimates

Estimator	(1)	(2)	(3)	(4)
	POLS	2FE	MG	CMG
Dependent variable is $\Delta {\ln\left(\text{HC}\right)}_{it}$				
Long‐run coefficients				
Log of life expectancy	1.371***	13.707	1.264	−0.426
	(0.089)	(12.291)	(0.849)	(0.286)
Short‐run coefficients				
Log of life expectancy	0.019	0.006	0.015	0.001
	(0.023)	(0.019)	(0.046)	(0.047)
Error correction coefficients				
ln(HC)_*i,t−1*_	−0.009***	−0.002	−0.026***	−0.083***
	(0.001)	(0.002)	(0.008)	(0.012)
Trend included	No	No	Yes	Yes
Year FE	Yes	Yes	No	No
Country FE	No	Yes	No	No
Observations	4029	4029	4029	4029
Diagnostics				
RMSE	0.01	0.01	0.00	0.00
CSD	0.12	0.67	0.00	0.35
CIPS	0.24	0.24	0.00	0.00

*Note*: This table reports empirical estimates of the long‐ and short‐run effects of life expectancy on human capital accumulation. The results are derived from estimating a dynamic model. The long‐run average coefficients are computed as $\theta_{i}=-\gamma_{i}/\rho_{i.}$ The standard errors of long‐run coefficients are calculated using the delta method. Robust standard errors in parentheses.

Abbreviation: RMSE, root mean squared error.

*** denotes statistical significance at the 1% level.

### 4.2 Omitted variables bias

Another major concern relates to potential omitted variable bias. It is important to highlight that the baseline findings are based on estimating a cointegrating vector. By examining residual‐based diagnostic tests, I rely on the CMG estimates with a stationary error term to draw statistical inference on the relationship between life expectancy and human capital. This helps mitigate potential bias induced by model misspecification. The conventional wisdom in estimating a cointegrating vector is that the estimates are insensitive to omitted stationary covariates (Stock, 1987). Suppose that if an important non‐stationary factor were to be omitted in the regression, it would be included in the error term. Hence, the unobserved components of the model would follow a non‐stationary process, making it difficult to establish cointegration (Everaert, 2011). It also follows from estimating a cointegrating vector that the estimates of a small set of non‐stationary variables will be robust to adding an extended set of control variables (Juselius, 2006, p. 11). This is in contrast to standard regression analyses in which the inclusion of control variables may drastically alter the results. For these reasons, it is common to specify relatively parsimonious models under a cointegration approach.^8^ To further address the above concern, I allow other important determinants of human capital accumulation to enter the benchmark model; the results are presented in Tables 10 and 11. More specifically, I follow Hansen (2013b) to control for log of GDP per capita and log of the infant mortality index, taken from the World Bank's World Development Indicators (http://wdi.worldbank.org/). However, the estimated effect of life expectancy on human capital remains insensitive to incorporating additional controls in the regression. It is noteworthy that all the models with additional controls do not pass the CSD test (Tables 10 and 11). For this reason, the baseline model specification is preferred due to the absence of model misspecification.

**TABLE 10:** The effect of life expectancy on human capital, controlling for GDP per capita

Estimator	(1)	(2)	(3)	(4)	(5)	(6)
	POLS	2FE	MG	CDMG	CMG	AMG
Log of life expectancy	0.819***	0.290***	0.140	0.452***	0.041	0.107
	(0.032)	(0.026)	(0.107)	(0.134)	(0.074)	(0.098)
Log of GDP per capita	0.103***	0.022***	−0.001	−0.001	0.025***	0.017*
	(0.003)	(0.004)	(0.016)	(0.018)	(0.008)	(0.010)
Year FE	Yes	Yes	No	No	No	No
Country FE	No	Yes	No	No	No	No
Trend included	No	No	Yes	No	Yes	Yes
Observations	3937	3937	3937	3937	3937	3937
Number of countries	78	78	78	78	78	78
Diagnostics						
RMSE	0.15	0.06	0.02	0.03	0.01	0.01
CSD	0.00	0.10	0.00	0.00	0.00	0.00
CIPS	1.00	1.00	0.00	1.00	0.00	0.00
AR (1)	0.00	0.00				
AR (2)	0.00	0.00				

*Note*: This table reports empirical estimates of the effect of life expectancy on human capital accumulation across countries controlling for log of income per capita. Robust standard errors in parentheses.

Abbreviations: CDMG, cross‐sectional demeaned mean group; RMSE, root mean squared error.

*** and * denote statistical significance at the 1% and 10% levels, respectively.

**TABLE 11:** The effect of life expectancy on human capital, controlling for child health

Estimator	(1)	(2)	(3)	(4)	(5)	(6)
	POLS	2FE	MG	CDMG	CMG	AMG
Log of life expectancy	0.518***	0.241***	0.047	−0.195*	−0.015	0.099
	(0.036)	(0.026)	(0.125)	(0.115)	(0.106)	[0.113]
Log of infant mortality rate	−0.196***	−0.032***	−0.067***	−0.006	−0.036**	−0.069***
	(0.005)	(0.006)	(0.021)	(0.010)	(0.018)	[0.022]
Year FE	Yes	Yes	No	No	No	No
Country FE	No	Yes	No	No	No	No
Trend included	No	No	Yes	No	Yes	Yes
Observations	3994	3994	3994	3825	3994	3994
Number of countries	78	78	78	77	78	78
Diagnostics						
RMSE	0.15	0.06	0.02	0.02	0.01	0.01
CSD [*p*‐value]	0.00	0.09	0.00	0.00	0.00	0.00
CIPS [*p*‐value]	1.00	1.00	0.00	1.00	0.00	0.00
AR (1) [*p*‐value]	0.00	0.00				
AR (2) [*p*‐value]	0.00	0.00				

*Note*: This table reports empirical estimates of the effect of life expectancy on human capital accumulation across countries controlling for log of infant mortality rate. Robust standard errors in parentheses.

Abbreviations: CDMG, cross‐sectional demeaned mean group; RMSE, root mean squared error.

***, ** and * denote statistical significance at the 1%, 5% and 10% levels, respectively.

### 4.3 Reverse causation

As mentioned previously, a key challenge with identification stems from reverse causation. It is argued that educated people tend to enjoy higher income and better resources, which contribute to health improvements. In addition, higher levels of education, on average, are associated with better information and knowledge, potentially leading to healthier lives. Addressing this concern, previous studies attempt to isolate plausibly exogenous sources of variation in life expectancy that help explain human capital accumulation. For example, Acemoglu and Johnson (2007) and Hansen (2013b) employ long differences in life expectancy between 1940 and 2000 led by the onset of modern health technologies as a plausibly exogenous instrumental variable (IV) for life expectancy. However, this empirical strategy is not applicable in the current study as I attempt to exploit within‐country variations in the data.^9^

To address plausible concerns about reverse causation, I employ an inverse genetic distance‐weighted average of life expectancy (*LE_Gdistw*) as an IV for LE. My empirical strategy exploits the fact that health technologies and knowledge transcend national borders and such spillovers decay with cultural distance between countries. Using the genetic distance index of Spolaore and Wacziarg (2009), Hansen (2013a) indicates that countries that are genetically distant to the world frontier of health innovations tend to suffer from poorer population health due to greater cultural and biological impediments to the international dissemination of health technologies and knowledge. It has been established that significant health improvements over the last decades are attributed to the worldwide diffusion of health innovations (Cutler et al., 2006; Preston, 1975; Soares, 2007). It follows from this line of reasoning that health outcomes in other countries are relevant for explaining the variation in a country's population health. Consistent with Hansen (2013a), I assume that such spatial dependence in national health status decreases with genetic distance—a measure of long‐term relatedness between countries. The underlying idea is that genetic distance, associated with the length of time elapsed since two societies were separated from a common ancestor, captures the divergence in various inter‐generationally transmitted human characteristics (e.g., norms, values, beliefs, predispositions, preferences, cultures, and languages), thereby undermining the cross‐border dissemination of technologies and institutions (Spolaore & Wacziarg, 2009, 2016). Therefore, countries are more likely to learn, emulate, and adopt health technologies developed in their genetically proximate counterparts. This lends support to the relevance of the IV. The construction *LE_Gdistw* of can be expressed as follows:

$$LE\_Gdistw_{it}=\frac{\sum_{j\neq i}\frac{1}{Gdist_{ij}}LE_{jt}}{\sum_{j\neq i}\frac{1}{Gdist_{ij}}}$$

where $Gdist_{ij}$ is the genetic distance between countries i and j provided by Spolaore and Wacziarg (2009). $LE\_Gdistw_{it}$ is the inverse genetic distance‐weighted average of life expectancy in other countries.

The exogeneity condition requires that *LE_Gdistw* affects HC exclusively through shaping the variation in LE. However, this assumption cannot be tested because the disturbance terms are unobserved. To alleviate possible violations of the exclusion restrictions, I attempt to separate the cross‐border diffusion of health outcomes from the international dissemination of productivity and education. To this end, I control for the inverse genetic distance‐weighted averages of income per capita and HC. On this basis, *LE_Gdistw* arguably has no direct influence on human capital accumulation except through shaping population health. This approach is consistent with Acemoglu et al. (2019) who exploit regional averages of democratization as an IV for democracy in growth regressions. It is worth emphasizing that I rely on genetic distance to capture relatedness between countries rather than using geographic distance/proximity. It is observed that many geographically close countries differ substantially in cultures, histories, or languages. For instance, New Zealand and Australia are culturally distant to Fiji and Papua New Guinea—their neighbors, and close to Western Europe. In contrast to geographic distance, genetic distance is developed based on the differences in the allele distributions, predetermined since two societies departed from a common ancestor (Ashraf & Galor, 2018; Spolaore & Wacziarg, 2009). Hence, it offers an internationally comparable measure of long‐term relatedness between contemporary countries. Furthermore, the adoption of *LE_Gdistw* as the IV for LE mitigates concerns about deviations from the exogeneity condition, compared to using regional averages of LE or geographic distance‐weighted measures. In particular, this approach helps distinguish between the international diffusion of health technologies and knowledge, and regional economic spillovers, thereby providing some support for the validity of the exclusion restrictions.

Table 12 presents IV estimates of the impact of life expectancy on human capital accumulation. In line with the baseline analysis, I first report the POLS and 2FE estimates in columns (1) and (2) of Table 12. The results reveal that the plausibly exogenous part of life expectancy has a positive and statistically significant influence on human capital accumulation. I follow Andrews et al. (2019) to report the first‐stage *F*‐statistic of excluded instruments of Olea and Pflueger (2013). In column (2), the *F*‐statistic is smaller than the conventional threshold of 10, which raises concerns about weak instrument bias. To rule out this possibility, I construct identification‐robust Anderson‐Rubin confidence intervals that provide reliable inference on the relationship between LE and HC regardless of the relevance of the IV, as suggested by Andrews et al. (2019). Zero is excluded from these intervals, which is suggestive of the statistically significant impact of LE on HC. Overall, the homogeneous parameter IV estimates reported in Table 12 are consistent with the benchmark findings.

**TABLE 12:** IV estimates of the effect of life expectancy on human capital

Estimator	(1)	(2)	(3)	(4)
	POLS‐IV	2FE‐IV	MG‐IV	CMG‐IV
Panel A. Second‐stage estimates. Dependent variable is log of human capital index				
Log of life expectancy	1.056***	2.569**	−3.440	−3.940
	(0.094)	(1.022)	(2.243)	(2.763)
*Income_Gdistw*	0.013***	0.009	0.005	0.021
	(0.005)	(0.008)	(0.067)	(0.018)
*HC_Gdistw*	0.391***	−0.536	0.277	12.490
	(0.048)	(0.584)	(2.389)	(12.207)
Panel B. First‐stage estimates. Dependent variable is log of life expectancy				
*LE_Gdistw*	0.728***	0.120***		
	(0.044)	(0.042)		
Year FE	No	Yes	No	No
Country FE	No	Yes	No	No
Trend included	No	No	Yes	Yes
Observations	3848	3848	3848	3848
Number of countries	74	74	74	74
First‐stage *F*‐statistic	274.93	8.20		
Anderson‐Rubin confidence interval	[0.88, 1.23]	[1.46, 8.13]		
Diagnostics				
RMSE	0.18	0.13	0.09	0.06
CSD	0.00	0.003	0.58	0.08
CIPS	0.01	0.81	0.00	0.00
AR (1)	0.00	0.01		
AR (2)	0.00	0.01		

*Note*: This table reports instrumental variable (IV) estimates of the effect of life expectancy on human capital accumulation across countries. In particular, the results capture the impact of the plausibly exogenous component of life expectancy, isolated by an inverse genetic distance‐weighted average of life expectancy in other countries, on human capital index. Robust standard errors in parentheses.

Abbreviation: RMSE, root mean squared error.

*** and ** denote statistical significance at the 1% and 5% levels.

Turning to columns (3) and (4) of Table 12, I account for UCFs and parameter heterogeneity using the MG and CMG estimators. Baltagi et al. (2019) demonstrate that the common factor approach helps control for UCFs even under the presence of endogenous regressors. Furthermore, the cross‐sectional mean of parameters can be consistently estimated by the mean‐group type estimators with endogenous variables (MG‐IV) (Baltagi et al., 2019; Feng, 2020).^10^ Accordingly, the estimated coefficient on LE is imprecisely estimated at conventional levels of statistical significance when I account for UCFs and parameter heterogeneity. As suggested by the residual‐based diagnostic tests, the preferred estimates are the CMG‐IV results, which are consistent with the main findings. This helps rule out the possibility that the core results are driven by failure to control for reverse causation.

## 5 CONCLUSION

This paper revisits conventional wisdom in the long‐term comparative development literature asserting that increased life expectancy strengthens incentives for acquiring better human capital skills. This channel of influence is often regarded as a key mechanism through which life expectancy helps shape worldwide income differences. Hence, a wealth of literature has explored the relationship between longevity and education from both theoretical and empirical perspectives. Most empirical studies lend strong evidence to the significant positive effect of health improvements on human capital accumulation. By contrast, Acemoglu and Johnson (2007) empirically establish that rising longevity has no statistically significant or even negative effect on human capital. Therefore, the existing literature offers mixed findings when it comes to estimating the educational returns to health improvements. Against this backdrop, this paper exploits several panel estimators and re‐examines the health‐education relationship by explicitly accounting for UCFs and parameter heterogeneity. I demonstrate that these econometric issues are far from trivial even when the objective lies exclusively in estimating the influence of life expectancy on human capital.

More specifically, I find that life expectancy has a positive and statistically significant impact on the accumulation of human capital when using conventional panel estimators that assume parameter homogeneity and cross‐sectional independence. However, I argue that these estimates provide an invalid basis for statistical inference because they are derived from estimating misspecified models, evidenced by the serially correlated, non‐stationary, and cross‐sectional dependent residuals of the econometric models. In addition, I adopt the mean‐group type estimators under a common factor framework to control for UCFs and parameter heterogeneity. The heterogeneous parameter estimates reveal that the impact of rising longevity on education is statistically insignificant at conventionally accepted levels when I account for UCFs and parameter heterogeneity. My findings indicate that addressing CSD and parameter heterogeneity is central to securing reliable inference on the relationship between life expectancy and human capital.

An important implication derived from my findings is that UCFs and parameter heterogeneity cannot be ignored when estimating the causal effect of life expectancy on human capital accumulation. While incorporating UCFs in econometric models is plagued with various difficulties as argued earlier, the main results suggest that the common factor approach developed by Pesaran (2006) offers a practical solution to addressing these concerns. Furthermore, the main empirical exercises adopted within this paper place emphasis on the within‐country variation of data when it comes to measuring the influence of health capital on human capital accumulation. For this reason, understanding the health‐education nexus critically requires attention to heterogeneity across countries. Indeed, it is implausible to assume that the educational returns to health improvements, say, in the United States and Sierra Leone, are homogeneous. In this regard, a potential avenue for future research is to explore determinants and patterns of such heterogeneity in the effect of health improvements on human capital accumulation, or, more generally, economic performance. In this regard, subnational analyses promisingly improve our understanding of the heterogeneous importance of health improvements in driving educational attainment across the world.

## CONFLICT OF INTEREST

The author declares that there is no conflict of interest that could be perceived as prejudicing the impartiality of the research reported.

## Supporting information

Supplementary Material 1

## Data Availability

The data that support the findings of this study are available from the author upon reasonable request.

## ENDNOTES

^1^
The well‐known horizon channel posits that an individual's decision to continue in school depends on the future survival rate. In particular, the cost of obtaining knowledge is incurred when one is in school while the returns to education accrue after graduating. The incentives of investing in education, therefore, critically rely on future gains from education because the cost is often taken for granted when one decides to accumulate human capital. For this reason, a lower adult mortality rate acts as a catalyst for improving the quality of human capital (Kalemli‐Ozcan, 2002; Kalemli‐Ozcan et al., 2000; Soares, 2005).

^2^
The positive association between longevity and human capital is also supported by several micro‐level studies (Bleakley, 2009, 2018; Bleakley & Lange, 2009; Jayachandran & Lleras‐Muney, 2009; Lucas, 2010; Oster et al., 2013; Soares, 2006).

^3^
For example, Hansen (2013b) regresses years of schooling on life expectancy using a so‐called long difference panel comprising of only two dates in 1940 and 1980 (or 2000). In a sensitivity test, he also estimates 10‐year averaged panel data models.

^4^
A recent study by Kotschy (2021) exploits exogenous changes in life expectancy in the US driven by medical innovations in treating cardiovascular diseases in the 1960s, and establishes that health improvements contribute to higher levels of income from 1960 to 2000 through raising educational attainment. To the extent that the impact of health on education is heterogenous across countries, subnational analyses promisingly provide important insights into the health‐education relationship, as evidenced by the findings of Kotschy (2021). The aim of this paper, however, is to re‐examine existing studies that provide the cross‐country average effect of health on educational attainment. In this regard, my findings complement and motivate subnational analyses, such as Kotschy (2021).

^5^
The human capital index provided by the PWT is constructed based on the measure of years of schooling of Barro and Lee (2013), considering returns to education. In particular, the calculation puts more weight on lower levels of education. This helps reduce underestimation of the quality of human capital in developing economies. Another advantage of using this index lies in its broadest coverage of years, which is particularly relevant for the estimation strategies of this study.

^6^
I follow the suggestion of an anonymous reviewer to estimate panel VAR models. Online Appendix Figure A1 depicts the impulse responses with 95% confidence intervals. In response to the shock in life expectancy, human capital increases; this impact is also statistically significant as zero is excluded from the probability band. This is consistent with the homogeneous parameter estimates reported in Table 5.

^7^
The results, available on request, are insensitive to including further lags into the dynamic model.

^8^
Existing studies exploring the health‐education nexus also set up very parsimonious cross‐country OLS regressions (Acemoglu & Johnson, 2007; Cervellati & Sunde, 2015; Hansen, 2013b; Hazan, 2012; Zhang & Zhang, 2005).

^9^
The adoption of internal instruments (e.g., lags of life expectancy) as instrumental variables for current life expectancy within a system GMM framework of Blundell and Bond (1998) may provide an invalid basis for statistical inference due to possible deviations from the exogeneity condition. This is attributed to persistence in the impact of life expectancy on human capital accumulation over time. Furthermore, the results can suffer from weak instrument bias (Bazzi & Clemens, 2013).

^10^
When using heterogeneous panel estimators, there is no single linear first‐stage regression to report. This is because I conduct IV regressions for each individual country and compute an average of the national estimates of the effect of life expectancy on human capital. When using the MG and CMG estimators, *LE_Gdistw*, on average, has a positive and statistically significant impact on LE, which lends support to the relevance of the IV.
