# Supplementary material for: Life expectancy and human capital: New empirical evidence
Source: Health Econ. 2022 Oct 31;32(2):395–412. doi: 10.1002/hec.4626 (PMC10092450; doi:10.1002/hec.4626)
Supplement: Supplementary file 1 — Supplementary Material 1 [file HEC-32-395-s001.docx]

**Online Appendix for “Life expectancy and human capital: New empirical evidence”**

**Appendix A. List of countries**

| High-income countries |  | Middle- and low-income countries |
| --- | --- | --- |
| Australia, Austria, Belgium, Canada, Chile, Denmark, Finland, France, Greece, Hong Kong SAR of China, Ireland, Italy, Japan, Korea Republic, Netherlands, New Zealand, Norway, Panama, Portugal, Singapore, Spain, Sweden, Switzerland, Trinidad and Tobago, United Kingdom, United States, and Uruguay. |  | Algeria, Argentina, Bangladesh, Bolivia, Brazil, Cameroon, China, Colombia, Congo Republic, Costa Rica, Cote d'Ivoire, Dominican Republic, Ecuador, Egypt, El Salvador, Ethiopia, Gabon, Ghana, Guatemala, Honduras, India, Indonesia, Iran, Jamaica, Jordan, Kenya, Madagascar, Malaysia, Mauritania, Mexico, Morocco, Mozambique, Myanmar, Nicaragua, Nigeria, Pakistan, Paraguay, Peru, Philippines, Romania, Senegal, South Africa, Sri Lanka, Syria, Tanzania, Thailand, Togo, Tunisia, Turkey, Venezuela, Zambia, and Zimbabwe. |

**Appendix B. Parameter homogeneity test**

Pesaran and Yamagata (2008) develop a test of parameter homogeneity based on the Swamy’s (1970) test statistic in large panels (the test requires both $T$ and $N$ to be large). The null hypothesis of the test is parameter homogeneity, which implies that the estimated effect is similar across cross-sectional units.

The delta test statistic is computed as:

$$\tilde{\Delta}=\sqrt{N}\left( \frac{N^{-1}\tilde{S}-k}{\sqrt{2k}} \right)$$

The bias-adjusted delta test statistic is shown as:

$$\tilde{\Delta}_{adj}=\sqrt{N}\left( \frac{N^{-1}\tilde{S}-E\left( \tilde{z}_{iT} \right)}{\sqrt{Var\left( \tilde{z}_{iT} \right)}} \right)$$

in which $\tilde{S}$ corresponds to the modified version of Swamy’s (1970) test statistic, which is based on the dispersion of cross-sectional unit specific estimates from a weighted fixed-effects pooled estimator. The mean and variance are represented by $E\left( \tilde{z}_{it} \right)=k$ and $Var\left( \tilde{z}_{iT} \right)=2k(T-k-1)/(T+1).$ These test statistics have an asymptotically standard normal distribution as the cross-sectional and time-series dimensions go to infinity $\left( \left( N,T \right)\to\infty\right)$ such that $\frac{\sqrt{N}}{T^{2}}\to0$. **Appendix C. Cross-sectional dependence test**

To find evidence of cross-sectional (in)dependence, I implement the test for weak cross-sectional dependence in large panels developed by Pesaran et al. (2004) and Pesaran (2015). The test statistic is computed using the average of the pairwise correlations of the OLS residuals ($\hat{\rho}_{ij}$) as follows:

$${CSD}_{NT}=\sqrt{\frac{2T}{N(N-1)}}\left( \sum_{i=1}^{N-1} \sum_{j=i+1}^{N} \hat{\rho}_{ij} \right)$$

The CSD test statistics under the null of error cross-sectional independence are asymptotically distributed as $N\left( 0,1 \right)$. Rejecting the null provides evidence of strong cross-sectional dependence.

**Appendix D. Accounting for UCFs under a common factor framework**

A key distinguishing feature of this study rests upon estimating heterogeneous panel data models under a common factor framework developed by Pesaran (2006). As argued earlier, this approach helps account for UCFs and parameter heterogeneity, thus providing a basis for reliable inference on the effect of life expectancy on human capital accumulation across countries. Therefore, I provide additional details about the common factor approach of Pesaran (2006). More specifically, I demonstrate that ignoring UCFs may yield biased and inconsistent estimates of the relationship between life expectancy and human capital. In addition, I show how potential bias induced by the presence of UCFs can be mitigated by introducing cross-sectional averages of variables in the benchmark model, following Pesaran (2006).

Consistent with the above discussion, the existence of UCFs, if not accounted for properly, may invalidate statistical inference if they are correlated with both life expectancy at birth and the human capital index. The underlying intuition can be demonstrated by the following equations:

${ln(HC)}_{it}=\beta_{i}{\ln(LE)}_{it}+\mu_{i}+\tau_{i}f_{t}+\varepsilon_{it}$ [3]

${ln(LE)}_{it}=\varphi_{i}+\lambda_{i}f_{t}+\epsilon_{it}$ [4]

Solving for the common factor ($f_{t}$) in Eq. [4] and substituting it into Eq. [3], we obtain the following equation:

${\ln\left( HC \right)}_{it}=\beta_{i}{\ln\left( LE \right)}_{it}+\mu_{i}+\tau_{i}{\lambda_{i}}^{-1}\left( {\ln\left( LE \right)}_{it}-\varphi_{i}-\epsilon_{it} \right)+\varepsilon_{it}=\underset{\omega_{i}}{\underbrace{\left( \beta_{i}+\tau_{i}{\lambda_{i}}^{-1} \right)}}{\ln(LE)}_{it}+\underset{\phi_{i}}{\underbrace{\mu_{i}-\tau_{i}{\lambda_{i}}^{-1}\varphi_{i}}}+\underset{\psi_{it}}{\underbrace{\varepsilon_{it}-\tau_{i}{\lambda_{i}}^{-1}\epsilon_{it}}}=\omega_{i}{\ln\left( LE \right)}_{it}+\phi_{i}+\psi_{it}$ [5]

It follows from the above mathematic demonstration that estimating Eq. [3] without controlling for UCFs ($f_{t}$) yields biased and inconsistent estimates of $E\left[ \beta_{i} \right]=\beta$ because $\omega_{i}=\beta_{i}+\tau_{i}{\lambda_{i}}^{-1}\neq\beta_{i}$. For this reason, the parameter coefficient ($\beta$) is not identified.

Next, the bias induced by the potential correlation between UCFs and life expectancy can be addressed by using cross-sectional averages as proposed by Pesaran (2006). From Eq. [3], the cross-sectional averages can be given by:

$\bar{{ln(HC)}_{t}}=\bar{\beta}\bar{{\ln\left( LE \right)}_{t}}+\bar{\mu}+\bar{\tau} f_{t}$ [6]

where the disturbance term ($\varepsilon_{it}$) is omitted from Eq. [6], assuming that $E\left[ \varepsilon\right]=0$. The common factor can be given by working out it from Eq. [6] as follows:

$f_{t}=\bar{\tau}^{-1}\left( \bar{{ln(HC)}_{t}}-\bar{\beta}\bar{{\ln\left( LE \right)}_{t}}-\bar{\mu} \right)$ [7]

Inserting Eq. [7] into [3], we obtain:

${\ln\left( HC \right)}_{it}=\mu_{i}-\tau_{i} \bar{\tau}^{-1}\bar{\mu}+\beta_{i}{\ln\left( LE \right)}_{it}+\tau_{i} \bar{\tau}^{-1}\bar{{\ln\left( HC \right)}_{t}}-\tau_{i} \bar{\tau}^{-1}\bar{\beta}\bar{{\ln\left( LE \right)}_{t}}+\varepsilon_{it}= \mu_{i}+\beta_{i}{\ln\left( LE \right)}_{it}+c_{1i}\bar{{ln(HC)}_{t}}+c_{2i}\bar{{\ln\left( LE \right)}_{t}}+\varepsilon_{it}$ [8]

From Eq. [8], we can obtain asymptotically unbiased and consistent estimates of $E\left[ \beta_{i} \right]=\beta$ even when UCFs are correlated with life expectancy at birth as suggested by Pesaran (2006). Importantly, introducing cross-sectional averages of variables in the baseline model does not require priori knowledge about the underlying relationship between UCFs and the main variable of interest (life expectancy), which is supposed to be unknown. By doing that, we address several shortcomings incurred when incorporating UCFs as controls in conventional regression analysis, as discussed earlier. Furthermore, UCFs may arguably exert heterogeneous impacts on variables in Eq. [3] ($\tau_{i}$), the identification above attempts to control for this by allowing for the effect of cross-sectional averages ($\bar{{ln(HC)}_{t}}$ and $\bar{{\ln\left( LE \right)}_{t}}$) to differ across countries. Therefore, the effect of UCFs ($f_{t}$) is implicitly allowed to vary across the world. Overall, the above mathematical expressions illustrate that the common factor framework of Pesaran (2006) can explicitly account for UCFs and their heterogeneous effect by incorporating cross-sectional averages of variables in regression analysis.

**Appendix E. Common correlated effect pooled estimator (CCEP)**

The common factor approach can be applied to both homogeneous and heterogeneous parameter panel estimators (Table 1). For the CCEP, Eq. [8] is augmented by multiplying cross-sectional averages by $N$ country dummies ($D_{j}$). The model can be specified as follows:

${\ln\left( HC \right)}_{it}=\mu_{i}+\beta{\ln\left( LE \right)}_{it}+\sum_{j=1}^{N} c_{1i}\left( \bar{{\ln\left( HC \right)}_{t}}D_{j} \right)+\sum_{j=1}^{N} c_{2i}\left( \bar{{\ln\left( LE \right)}_{t}}D_{j} \right)+\varepsilon_{it}$ [9]

**Appendix F. Augmented mean group estimator (AMG)**

Another widely used heterogeneous panel estimation method that considers the presence of UCFs is the augmented mean group estimator developed by Bond and Eberhardt (2009). While Pesaran (2006) proposes the use of cross-sectional averages to deal with CSD, Bond and Eberhardt (2009) control for UCFs by introducing a “common dynamic process” in the country regression. In particular, the implementation follows a two-step process. The first-step regression is based on estimating an equation in first differences using the pooled regression to obtain the estimated coefficients for $T-1$ year dummies, as demonstrated in Eq. [10]. The main intuition behind this empirical exercise is to capture the levels-equivalent mean evolution of UCFs across countries. The second-step regression involves estimating the baseline mean group estimation but incorporating the year dummy coefficients computed in the first-step regression, as shown in Eq. [11]. This is motivated by an assumption that UCFs form part of the country-specific cointegrating relationship (Pedroni, 2007). The mathematic expressions can be given as follows:

${\Delta\ln\left( HC \right)}_{it}=\beta_{i}\Delta{\ln\left( LE \right)}_{it}+\sum_{t=2}^{T} c_{t}\Delta D_{t}+\varepsilon_{it}$ [10]

${\ln\left( HC \right)}_{it}=\beta_{i}{\ln\left( LE \right)}_{it}+\mu_{i}+c_{i}t+d_{i} \hat{c_{t}}+\varepsilon_{it}$ [11]

where $\hat{c_{t}}$ is the estimated coefficients of $T-1$ year dummies obtained from estimating Eq. [10]. This is allowed to vary across countries in Eq. [11] ($d_{i}$). The mean group estimator of Pesaran and Smith (1995) is employed to estimate Eq. [11]. Therefore, the estimated effect is computed as the average of $N$ conventional country OLS regressions ($\hat{\beta}_{AMG}=N^{-1}\sum_{i} \hat{\beta}_{i}$).

**Appendix G. Additional results**

**Table A1. Summary statistics**

| Variables | Observations | Mean | Std. deviation | Min | Max |
| --- | --- | --- | --- | --- | --- |
| *Log of human capital index* | 4,108 | 0.701 | 0.335 | 0.016 | 1.320 |
| *Log of life expectancy* | 4,108 | 4.175 | 0.169 | 3.648 | 4.434 |
| *Log of human capital index in FD* | 4,029 | 0.010 | 0.006 | -0.007 | 0.043 |
| *Log of life expectancy in FD* | 4,029 | 0.005 | 0.006 | -0.031 | 0.044 |
| *Log of human capital (demeaned)* | 4,108 | -2.62e-09 | 0.299 | -0.757 | 0.667 |
| *Log of life expectancy (demeaned)* | 4,108 | 1.60e-08 | 0.152 | -0.475 | 0.274 |
| *Log of GDP per capita* | 3,937 | 8.437 | 1.485 | 4.898 | 11.425 |
| *Log of infant mortality rate* | 3,994 | 3.375 | 1.105 | 0.588 | 5.273 |
| *LE_Gdistw* | 3,848 | 4.195 | 0.111 | 3.805 | 4.408 |
| *HC_Gdistw* | 3,848 | 0.743 | 0.218 | 0.109 | 1.231 |
| *Income_Gdistw* | 3,848 | 8.127 | 1.136 | 0.618 | 10.907 |

**
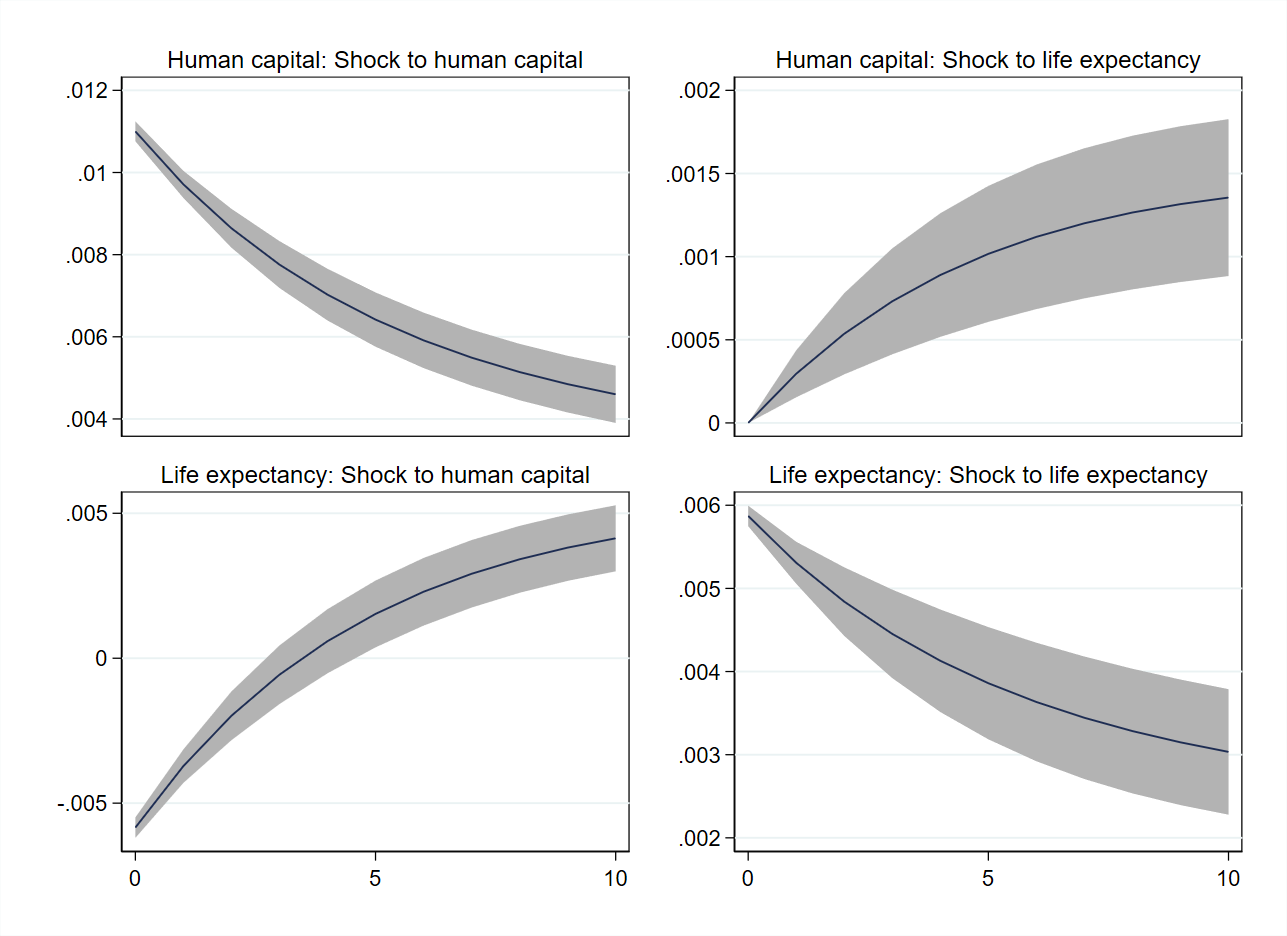
**

**Figure A1. Impulse responses for the VAR model**

**Appendix References**

Bond, S., & Eberhardt, M. (2009). *Cross-section dependence in nonstationary panel models: A novel estimator*. MPRA Paper 17692, University Library of Munich, Germany.

Pedroni, P. (2007). Social capital, barriers to production and capital shares: Implications for the importance of parameter heterogeneity from a nonstationary panel approach. *Journal of Applied Econometrics*, *22*(2), 429-451.

Pesaran, M. H. (2006). Estimation and inference in large heterogeneous panels with a multifactor error structure. *Econometrica*, *74*(4), 967-1012.

Pesaran, M. H. (2015). Testing weak cross-sectional dependence in large panels. *Econometric Reviews*, *34*(6-10), 1089-1117.

Pesaran, M. H., Schuermann, T., & Weiner, S. M. (2004). Modeling regional interdependencies using a global error-correcting macroeconometric model. *Journal of Business & Economic Statistics*, *22*(2), 129-162.

Pesaran, M. H., & Smith, R. (1995). Estimating long-run relationships from dynamic heterogeneous panels. *Journal of Econometrics*, *68*(1), 79-113.

Pesaran, M. H., & Yamagata, T. (2008). Testing slope homogeneity in large panels. *Journal of Econometrics*, *142*(1), 50-93.

Swamy, P. A. V. B. (1970). Efficient inference in a random coefficient regression model. *Econometrica*, *38*(2), 311-323.
